# Butyrylcholinesterase and 24 h-urinary copper excretion as compliance assessment in long-term treated Wilson’s disease

**DOI:** 10.3389/fneur.2025.1553573

**Published:** 2025-05-19

**Authors:** Harald Hefter, Max Novak, Dietmar Rosenthal, Sven G. Meuth, Tom Luedde, Philipp Albrecht, Christian J. Hartmann, Sara Samadzadeh

**Affiliations:** ^1^Department of Neurology, University of Düsseldorf, Düsseldorf, Germany; ^2^Department of Gastroenterology, University of Düsseldorf, Düsseldorf, Germany; ^3^Department of Neurology, Maria Hilf Clinics Mönchengladbach, Mönchengladbach, Germany; ^4^Charité–Universitätsmedizin Berlin, Freie Universität Berlin and Humboldt-Unverstät zu Berlin, Experimental and Clinical Research Center, Berlin, Germany; ^5^Institute of Regional Health Research and Institute of Molecular Medicine, University of Southern Denmark, Odense, Denmark; ^6^Department of Neurology, Slagelse Hospital, Slagelse, Denmark

**Keywords:** Wilson’s disease, butyrylcholinesterase, 24 h-urinary copper excretion, compliance, long-term treatment

## Abstract

**Background and aim of the study:**

Compliance is the most challenging aspect of long-term therapy in Wilson’s disease (WD). Evidence is presented that butyrylcholinesterase (CHE) can be used as a sensitive biomarker to detect compliance problems in long-term treated WD-patients.

**Methods:**

For the present retrospective, monocentric study demographical and treatment related data of 108 WD-patients (who had been treated at the Clinic of Neurology of the university hospital in Düsseldorf (Germany) between 2/2005 and 1/2021) were extracted from the charts. These patients underwent 2003 therapy control visits. The present study focuses on the analysis of three parameters of copper metabolism (serum levels of ceruloplasmin (CER-S), copper (CU-S) and the 24 h-urinary copper excretion (24 h-UCU)) and the serum levels of CHE (CHE-S). A patient was classified to be non-compliant when in his charts at least 8 24 h-UCU-values were found, and all his 24 h-UCU-values were larger than 60 μg/d (N-COM8-group). A patient was classified to be compliant when at least one of at least 8 24 h-UCU-values was lower than or equal to 60 μg/d (COM8-group).

**Results:**

CHE-S was significantly (*p* < 0.05) different between thus defined compliant or non-compliant patients. Neither CU-S nor CER-S nor calculated free (non-ceruloplasmin-bound) serum copper levels (NCC-S) were significantly different between the NCOM8- and COM8-group. Analysis of the area under the curve and sensitivity and specificity by means of ROC-curves underlined the sensitivity of CHE-S in contrast to the insensitivity of CU-S and CER-S to detect patients who had been classified as compliant/non-compliant on the basis of their 24 h-UCU-values.

**Conclusion:**

When compliance of WD-patients is classified on the basis of their 24 h-urinary copper excretion CHE-S is more sensitive to detect problems of non-compliance than serum levels of copper, of non-ceruloplasmin bound free copper or ceruloplasmin. Therefore, CHE-S may be used as an easy to determine further biomarker for compliance assessment in long-term treatment of WD in addition to 24 h-UCU.

## 1 Introduction

In Wilson’s disease (WD) mutations of the P-adenotriphosphate7Bprotease (ATP7B) encoding gene on chromosome 13q14.3-q21.1 (1–5) lead to disturbed synthesis of ATP7B which is necessary for the excretion of copper (CU) from the liver into the bile (6, 7) and the incorporation of copper into apo-ceruloplasmin to produce the main copper transport protein holo-ceruloplasmin (CER) in the hepatocytes (8). This causes accumulation of copper in the liver, followed by increased serum levels of loosely, non-ceruloplasmin bound copper (NCC-S) and finally a copper overload of the entire organism (9, 10).

Life-long medication with copper chelating agents and/or zinc preparations is necessary to prevent manifestation of WD in asymptomatic patients or to reduce symptoms when WD has already become manifest (9, 10). Under continuous medication laboratory findings and clinical symptoms may recover. Usually, recovery of gastrointestinal symptoms occurs more rapidly than recovery of neuropsychiatric symptoms (11). To achieve recovery or maintenance of a symptomatic state compliance of the WD-patients is absolutely necessary.

No relevant change of symptoms occurs after cessation of medication for a few days as during the collection of the 24 h-urine after a 48 h-cessation of medication for therapy monitoring (12). This implies that adherence to therapy is a minor aspect of compliance in WD in contrast to persistence to medication which is difficult to control. Usually, persistence to medication in WD is analyzed by structured interviews at the control visits (13), or on phone, or in the social media (14). Rates of admitted incompliance vary between 25 and 45% (12–15). It can be concluded that in clinical practice non-compliance is even more common in WD. Even complete cessation of medication has repeatedly been reported (16–18).

The gold-standard for therapy monitoring in WD is the 24 h-urinary copper excretion (24 h-UCU). In a study comparing 20 WD-patients who had been classified by interview to be compliant with 12 patients who had been classified to be non-compliant the 24 h-UCU measured after 48 h-cessation of medication was significantly higher and the clinical outcome was significantly worse in the non-compliant compared to the compliant patients (12).

However, for the exact determination of 24 h-UCU a variety of pitfalls exist. Some patients do not stop intake of their medication for 48hs before collection of the urine, take medication during collection, do not collect the urine completely over 24hs, and contaminate the urine sample by not using specially prepared vessels which can alter the result considerably (19, 20). Nevertheless, so far 24 h-UCU remains the gold standard of therapy monitoring in WD although it heavily relies on patient’s compliance (12). To date, there is no reliable single test, that can be used to monitor copper metabolism, proper treatment and compliance in clinical practice (12) and there is still a need for a cheap, simply to determine biomarker which is sensitive enough to detect non-compliance in WD.

In previous years butyrylcholinesterase has been described as a prognostic marker for a variety of diseases with liver impairment (21, 22) as liver dysfunction and damage in pesticide spraying farmers (23) and in patients with chronic (24) or acute (25) heart failure. CHE-S even correlates with the severity of COVID19-infection (26). In combination with albumine it may be used as a novel biomarker for hepatocellular carcinoma post hepatectomy (27).

In WD, it was recently demonstrated that CHE-S is a sensitive biomarker not only to distinguish between untreated WD-patients and normal subjects but also between untreated WD-patients and heterozygous gene carriers (28).

We therefore designed the present retrospective cross-sectional study. Following the recommendation that 24 h-UCU-values are a helpful tool for compliance measurement (12) a large cohort of long-term treated WD-patients was split-up into a compliant and a non-compliant subgroup by means of the 24 h-UCU-values. The hypothesis was tested that thus-defined non-compliant patients had lower CHE-S-values than compliant patients.

## 2 Methods

### 2.1 Patients

For the present combined retrospective and cross-sectional mono-centric study all charts of patients with movement disorders having been treated between 2/2005 and 1/2021 in the out-patient department of the Clinic of Neurology of the University hospital in Düsseldorf (Germany) were screened. Included were patients in whom (i) WD was diagnosed or confirmed at the University hospital in Düsseldorf, (ii) for whom at least one complete set of 34 biochemical parameters including a measurement of 24 h-UCU was available in the laboratory computer of the hospital, and (iii) who had had a control visit between Feb./2005 and Jan./2021.

Finally, 108 WD-patients were included. All of them gave informed consent for publication of their pseudonymized data. They underwent numerous (=2003) control visits. Demographical and treatment related data of these control visits were extracted from the charts of the patients and the hospital laboratory computer.

According to a general statement of the local ethics committee at the University of Düsseldorf, no special application is necessary for a pure retrospective study.

### 2.2 Analysis of four biochemical parameters (CER-S, CU-S, 24 h-UCU, CHE-S)

The present study focuses on the analysis of the three parameters of copper metabolism serum level of ceruloplasmin (CER-S), serum level of copper (CU-S) and 24 h-urinary copper excretion (24 h-UCU) as well as serum levels of pseudocholinesterase (CHE-S). All available values of the 2003 control visits were extracted for the present analysis.

### 2.3 Definition of compliance/non-compliance on the basis of NCC-S

In a first step mean CER-S (mg/dl) and mean CU-S (mg/l) were determined for each patient. For some serum levels of CER-S below 7 mg/dL the exact value had not been determined and was set to 6 mg/dL for the present analysis. The regression line between mean CER-S und mean CU-S was determined. Since 1 mg of intact CER-S usually transports 0.00315 mg copper (29), NCC-S (mg/l) per patient was determined using the following formula:

NCC-S (mg/l) = CU-S (mg/l) − 0.0315 CER-S (mg/dl).

A patient was classified to be non-compliant when mean NCC-S was larger than 0.1 mg/L (=10 μg/dL) and was classified to be compliant when NCC-S was lower than or equal to 0.1 mg/L. The N-COM-NCC-subgroup contained all thus-defined non-compliant and the COM-NCC-subgroup all compliant patients.

### 2.4 Definition of compliance/non-compliance on the basis of 24 h-UCU

According to inclusion criterium (ii) all patients had at least one 24 h-UCU-value. Patients with all 24 h-UCU-values ≤60 μg/d were classified as highly compliant (H-COM1-subgroup), patients with at least one 24 h-UCU-value >60 μg/d were classified as less compliant (L-COM1-subgroup), and patients with all 24 h-UCU-values >60 μg/d were classified as non-compliant (N-COM1-subgroup).

To take into account that CHE-S is low in untreated newly diagnosed patients, final analysis was based on 65 patients with at least eight 24 h-UCU-values (corresponding to at least 2 years of continuous treatment). These 65 patients were split-up into a non-compliant group (N-COM8-subgroup; *n* = 9; all 24 h-UCU values >60 μg/d) and a compliant group (COM8-group; *n* = 56; at least one 24-UCU-value ≤60 μg/d).

After this classification process for each of these 65 patients six parameters were selected for comparison between the N-COM8- and COM8-subgroup: the first CHE_S-value (IN-CHE-S), the last CHE-S-value (LA-CHE-S), mean CHE-S, mean CU-S, mean CER-S, and mean NCC-S.

Thereafter, an analysis of receiver operating characteristics ((ROC)-curves) was performed which of these parameters predicted the best the classification of the 65 WD-patients (with at least 8 24 h-UCU-values) into compliant (COM8-group) and non-compliant patients (N-COM8-group).

### 2.5 Statistics

Parameters of descriptive statistics as mean values (MVs) and standard deviations (SDs) were determined for demographical (age at recruitment (AGE), age at diagnose and onset of therapy (AGO)) and biochemical parameters (CER-S, CU-S, 24 h-UCU, CHE-S). Correlations were determined by means of the non-parametric rang correlation coefficient (RCC). For some parameter combinations (see Results section) also the regression line and the Pearson product moment correlation coefficient was calculated. T-testing (without the assumption of equal variance) was performed to detect differences between mean values of 6 parameters of the N-COM8- and the COM8-subgroup. Significance levels were corrected for multiple comparisons. ROC-curve analysis (determination of the area under the curve (AUC) and estimation of sensitivity and specificity for 5 parameters) was performed to test which parameter differentiated between non-compliant and compliant patients the best.

Determination of regression lines, RCCs, t-tests und ROC-curve analysis were part of the commercially available SPSS-statistics package (version 25; IBM, Armonk, United States).

## 3 Results

### 3.1 Demographical and treatment related data

The entire cohort of 108 WD-patients contained 63 (=58.3%) female and 45 (=41.7%) male patients. Mean age at diagnosis of WD (AGO) and onset of treatment was 22.4 years (SD: 10.1 years; range: 2.1–54.2 years). Mean age at recruitment (AGE) was 41.1 years (SD: 12.9 years; range: 13.9–75.8 years). Mean duration of treatment (DURT = AGE-AGO) was 18.8 years. (SD: 11.3 years; range: 0.4–54.6 years).

### 3.2 Analysis of four biochemical parameters (CHE-S, CU-S, CER-S, 24 h-UCU)

**Figure 1:**
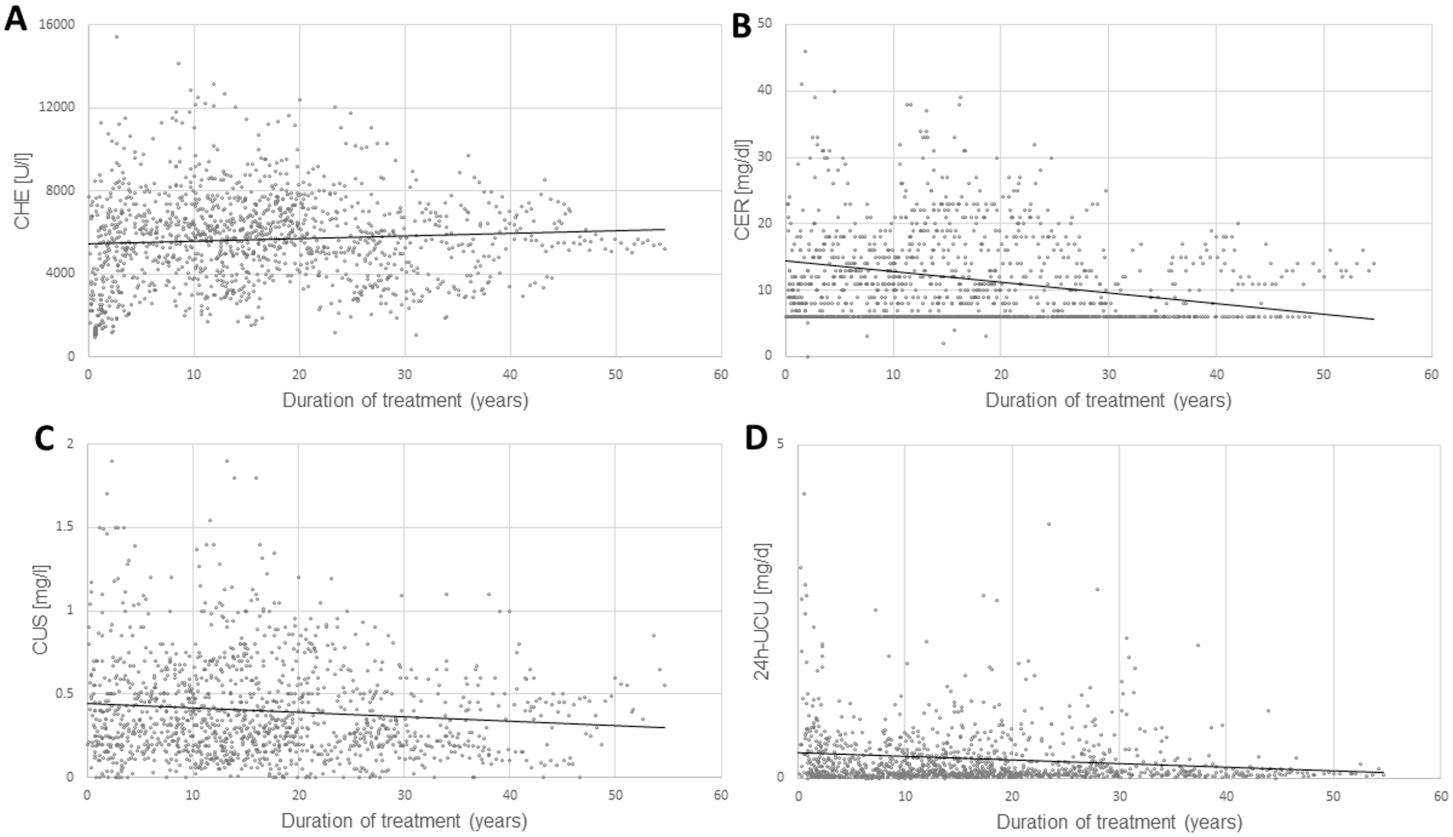
The temporal development of four laboratory parameters [serum levels of pseudocholinesterase (CHE-S)]. **(A)** Serum levels of ceruloplasmin (CER-S), **(B)** serum levels of copper (CU-S), **(C)** 24 h-urinary copper excretion (24 h-UCU), **(D)** from onset of therapy are presented together with the regression lines (dotted lines in **A–D**) between these 4 parameters and the duration of treatment. The variability of all 4 parameters is high, especially of 24 h-UCU, the gold- standard for therapy monitoring.

In Figure 1 all available data of the four parameters CHE-S (Figure 1A), CER-S (Figure 1B), CU-S (Figure 1C) and 24 h-UCU (Figure 1D) are presented together with the regression lines between these 4 parameters and duration of treatment (DURT). All four parameters showed a large variability. Minimal values (MIN), maximal values (MAX), and the mean values (MV) as well as the corresponding standard deviations (SD) of these four parameters as well as the corresponding normal ranges are presented in Table 1. Only the mean value of CHE-S was within the normal range.

**Table 1:** Minimal, maximal, and mean values and standard deviations of CHE-S, CU-S, CER-S and 24 h-UCU.

Parameters	MIN	MAX	MV	SD	Normal range
AGO (years)	2.1	54.2	22.4	10.1	n.a.
AGE (years)	13.9	75.8	41.1	12.9	n.a.
CHE-S (U/L)	964	15,400	5,622	2,169	5,320–12,920
CU-S (μg/dL)	9.9	192.5	40.1	29.4	82–139
CER-S (mg/dL)	2.0	140.0	10.9	7.9	20–60
24 h-UCU (μg/d)	9.9	15,000	303.5	660.6	≤60

MIN, minimal value; MAX, maximal value; MV, mean value; SD, standard deviation; AGO, age at diagnose; AGE, age at recruitment; CHE-S, serum level of cholinesterase; CU-S, serum level of copper; CER-S, serum level of ceruloplasmin; 24 h-UCU, 24 h-urinary copper excretion.

**Figure 2:**
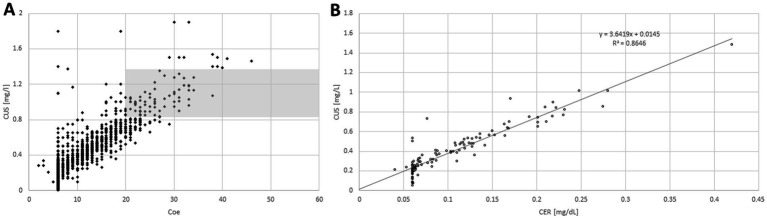
When individual serum levels of copper (CU-S; y-axis) were plotted against individual serum levels of ceruloplasmin (CER-S; x-axis; **A**) most of the pairs of individual values were lying outside of the combined normal range of CU-S and CER-S (gray area). When mean CU-S-value of a patient is plotted against the mean CER-S-value **(B)** a clear (highly significant, *p* < <0.001) linear relationship between CU-S and CER-S becomes obvious.

When these four parameters were correlated with AGO and AGE significant rang correlation coefficients were found for all four parameters (for details see Table 2). When these four biochemical parameters were correlated with each other, an (expected) highly significant (*p* < 0.001 × 10^−10^) correlation between CER-S and CU-S was found (see also Figures 2A,B). A highly significant (*p* < 0.876 × 10^−8^) correlation was also found between CHE-S and 24 h-UCU. But no correlation was found between CHE-S and CU-S or CER-R and no correlation between 24 h-UCU and CU-S and CER-S. The highly significant negative correlation between CHE-S and 24 h-UCU is a first hint that compliance or non-compliance defined on the basis of 24 h-UCU-values (as suggested in (12)) may also be detected by CHE-S.

**Table 2:** Rang correlation coefficients and *p*-values.

Parameters	AGO	AGE	CHE	CUS	CER	24 h-UCU
AGO	–	0.717	0.098	−0.083	−0.084	−0.111
AGE	<0.00001	–	0.098	0.110	0.136	−0.098
CHE-S	0.259 × 10^−3^	0.238 × 10^−3^	–	−0.035	−0.021	−0.176
CU-S	0.192 × 10^−2^	0.435 × 10^−4^	0.215	–	0.853	−0.007
CER-S	0.137 × 10^−2^	0.002 × 10^−4^	0.449	<0.00001 × 10^−8^	–	−0.022
24 h-UCU	0.693 × 10^−4^	0.410 × 10^−3^	0.896 × 10^−8^	0.817	0.470	–

AGO, age at diagnosis; AGE, age at recruitment; CHE-S, serum level of cholinesterase; CU-S, serum level of copper; CER-S, serum level of ceruloplasmin; 24 h-UCU, 24 h-urinary copper excretion. The upper part of the table contains the rang correlation coefficients (RCC) and the lower part the corresponding p-values. Significant correlations are highlighted.

When CU-S (y-axis) was plotted against CER-S (x-axis) most of the individual values (Figure 2A) were lying outside of the combined normal range of CU-S and CER-S (gray area in Figure 2A). When the mean value of the CU-S-values (ordinate) of a patient was plotted against the mean value of his CER-S-values (abszissa) a clear linear relationship (CUS = 0.3649*CER + 0.0145; *r* = 0.9298; *p* <0.001) between CU-S and CER-S became obvious (Figure 2B). Because the very small CER-S-values of some patients were set to 6 mg/dL (see Figure 2B) the regression line between CER-S- and CU-S-values was slightly steeper than expected according to the CU-S = 0.315*CER-S-relation mentioned in the methods.

### 3.3 Compliant and non-compliant patients determined on the basis of NCC

For each pair of CER-S and CU-S-values the free non-ceruloplasmin bound serum copper level (NCC-S; see Methods) was calculated and the mean NCC-S-value was determined. When compliance was defined in dependence on the mean NCC-value whether it was larger than 0.1 mg/L or lower than or equal to 0.1 mg/L the non-compliant patient subgroup (N-COM-NCC-group) comprised 42 (=38.9%) patients and the compliant subgroup (COM-NCC-group) 66 (=61.1%) patients. The copper transport capacity of CER-S is usually overestimated since its determination usually includes the inactive apo-CER-S. Therefore, NCC-S may become negative as was the case in 36 (=33.3%) patients of the present cohort. As a consequence, we did not further use the NCC-S-criterion (> 0.1 mg/L or ≤ 0.1 mg/L) to split-up the cohort into a non-compliant and compliant subgroup.

### 3.4 Compliant and non-compliant patients determined on the basis of 24 h-UCU

Following a previous recommendation (12) patients were also split-up according to their 24 h-UCU-values. A patient was classified to be non-compliant when all his 24 h-UCU-values were > 60 μg/d (N-COM1-group; n = 29 (=26.9%)), was classified to be less compliant when at least one 24 h-UCU-value was <60 μg/d (L-COM1-group; n = 79 (=73.1%)) and was classified to be highly compliant patients when all his 24 h-UCU-values were smaller than or equal to 60 μg/d (H-COM1-group; n = 9 (=8.3%)).

However, none of these 9 H-COM1-patients had more than seven 24 h-UCU-values. Thus, either these patients were newly diagnosed patients or had not collected the 24 h-urine over a longer time period. Since CHE-S is low in newly diagnosed patients (28) and usually increases during the first years of treatment (comp. Figure 1A), the final data analysis was only based on patients with at least eight 24 h-UCU-values. Nine non-compliant patients were detected with all 24 h-UCU-values being larger than 60 μg/d (N-COM8-group) and 56 compliant patients with at least one 24 h-UCU-value lower than or equal to 60 μg/d (COM8-group).

### 3.5 Comparison of CHE-S, CU-S, NCC-S, and CER-S in compliant and non-compliant patients

For each patient the initial CHE-S-value (IN-CHE-S) and the last CHE-S-value (LA-CHE-S) was selected and the mean CHE-S, the mean CU-S-, the mean CER-S- and the mean NCC-S-value were determined. Then mean values and standard deviations were calculated per compliance group (see Table 3). A tendency for a difference between the N-COM8- and the COM8-group was found for the initial and the last CHE-S-value. But after alpha-adjustment these differences were not significant anymore. The small differences of mean CU-S, mean CER-S and mean NCC-S between the two compliance subgroups were not significant. Only mean CHE-S showed a significant difference, even after alpha-adjustment (for details see Table 3). Thus, the hypothesis proposed in the introduction could be confirmed.

**Table 3:** Comparison of six parameters between the N-COM8- and the COM8-group.

		N-COM8-group		COM8-group		Significance level	
Parameter	Units	MV	SD	MV	SD	Before adj.	After adj.
IN-CHE-S	U/l	3,830	1828	5,509	2,448	0.0303	n.s.
LA-CHE-S	U/l	4,155	1945	6,102	1837	0.0179	n.s.
Mean CHE-S	U/l	4,139	1,250	5,893	1785	0.0027	0.016
Mean CU-S	mg/l	0.425	0.293	0.405	0.229	0.8531	n.s.
Mean CER-S	mg/l	11.36	6.24	10.85	5.54	0.8224	n.s.
Mean NCC-S	mg/l	0.062	0.109	0.041	0.081	0.6057	n.s.

MV, mean value; SD, standard deviation, IN-CHE-S, first available CHE-S-value of a patient; LA-CHE-S, last CHE-value of a patient; CHE-S, serum level of cholinesterase; CU-S, serum level of copper; CER-S, serum level of ceruloplasmin; NCC-S, calculated serum level of non-ceruloplasmin bound copper.

**Figure 3:**
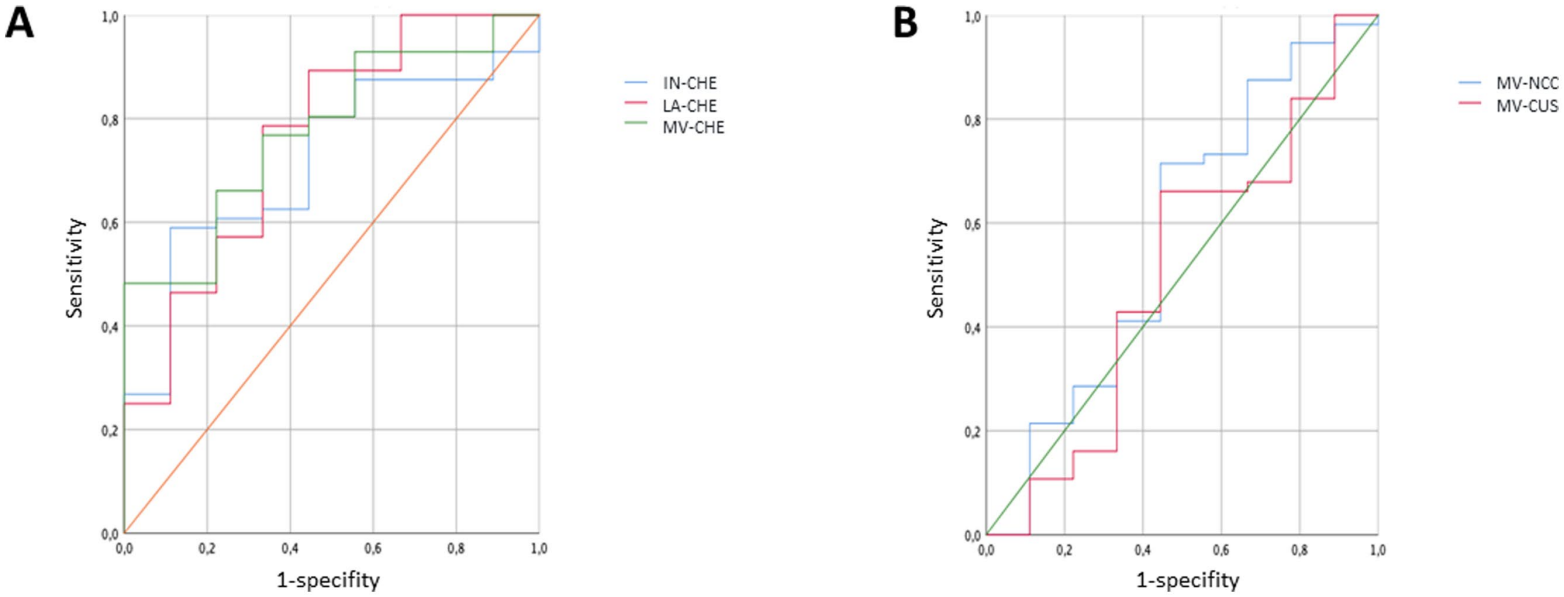
ROC-curves were determined for three CHE-S-measurements (IN-CHE-S, LA-CHE-S, mean CHE-S) in **(A)** and for mean CU-S and mean NCC-S in **(B)** when patients were classified as compliant/non-compliant patients according to their 24 h-UCU-values. The ROC-curves in **(A)** indicate a significant impact of the classification on the three CHE-S-parameters, whereas the ROC-curves of mean CU-S and mean NCC-S vary around the midline in **(B)**.

To analyze the sensitivity and the specificity to classify non-compliant and compliant patients correctly ROC-curves and the area under the curve (AUC) were determined for 5 parameters (IN- CHE-S, LA-CHE-S, mean CHE-S, mean CU-S, mean NCC-S). The largest AUC was observed for mean CHE-S (=0.776; *p* < 0.0083), followed by LA-CHE-S (=0.762; *p* < 0.0122) and followed by IN-CHE-S (=0.716; *p* < 0.0384) indicating a significant impact of the 24 h-UCU classification on these 3 CHE-S-parameters (see Figure 3A). The ROC-curves of mean CU-S (AUC = 0.496; *p* = 0.9697) and mean NCC-S (AUC = 0.427; *p* = 0.4822) varied around the midline in Figure 3B and indicated no relevant influence of the 24 h-UCU values on CU-S and NCC-S corresponding to the missing correlation mentioned above. Both sensitivity of mean CHE-S (0.768) and specificity (0.666) were low to detect non-compliant patients when compliance/non-compliance was defined on the basis of 24 h-UCU-values. Sensitivity and specificity of the other four parameters were even lower.

## 4 Discussion

### 4.1 General aspects of non-compliance in WD

When Walshe introduced D-penicillamine (DPA) for the treatment of WD in 1956 he revolutionized outcome perspectives for patients with WD (30). In the pre-DPA-era very painful BAL-injections had been used with little or no effect (31). But after some years of experience with DPA in WD it was realized that a fairly large percentage of WD-patients were non-compliant (16, 17, 32). WD-patients not only did not adhere to a rigid intake regimen but even stopped medication for days, weeks and even months with dangerous consequences. Fatal outcome or severe worsening after cessation of medication has been reported repeatedly (16–18).

Compliance can definitely be improved by reducing intake of medication to once per day (as is possible for tetramolybdate or trientine). But electronic devices which remind the patient to take his medication are also very helpful for compliance improvement.

In structured interviews during control visits, or on phone or social media the percentage of WD-patients who admit non-compliance varies between 25 and 45% world-wide (13–15). Our estimation of the rate of non-compliant WD-patients by means of NCC of 38.9% (42/108) and by means of 24 h-UCU-values of 26.9% (29/108) is fully compatible with these reported rates. Non-compliance, especially non-persistence to therapy is the main problem during long-term treatment of WD. Whether female WD-patients are more compliant than male WD-patients is an interesting question and will be analyzed in a separate study.

### 4.2 Compliance assessment by means of NCC

It has been mentioned that in non-compliant WD-patients NCC-S is high (>25 μg/dL) and is very low (<5 μg/dL) in cases of overtreatment (12, 33) (compare Results 3.3). Even negative values for NCC-S have been reported (29, 34–36) in 10 to 25% of the patients. These negative values result from an overestimation of serum levels of CER-S, when immunological methods are used which do not discriminate between apo- and holo-CER (20, 29). Dziezyc et al. (12) observed negative NCC-S-values in 12 of 32 patients (=37.5%). In our much larger cohort 35% of the patients had negative NCC-S-values. We therefore agree with the EASL that NCC-S (when determined by calculation from serum levels of copper and ceruloplasmin) is not recommended for diagnosis of WD (29, 33) and that NCC-S is of little value for therapy monitoring of WD.

But in principle, the determination of NCC-S is an excellent approach to derive a sensitive and specific biomarker for diagnosis and therapy monitoring in WD when a more sophisticated approach is used (20, 37, 38). When ultrafiltration is performed after a 1 h-incubation of a blood sample with EDTA the easy exchangeable fraction of serum copper can be determined (CUEXC) and the ratio (REC) between CUEXC and total serum copper (CUS) can be calculated. CUEXC and REC are promising biomarkers not only for the diagnosis of WD but also for therapy monitoring since they have a high sensitivity and specificity for WD (39) and correlate with clinical outcome (20, 39). Unfortunately, these parameters are not available for most WD-centers (20, 29) so far and are too expensive for routine therapy monitoring (20). But this may change in future.

### 4.3 Compliance assessment by means of 24 h-UCU-values

The 24 h-UCU is the gold standard of therapy monitoring in WD despite of a variety of short comings and pitfalls (see Introduction). In children or patients with neurological disorders or patients with incontinence the determination of the exact volume of the 24 h-urine may be difficult (20). In patients with renal dysfunction the value of the 24 h-UCU may be misleading (20, 40). Furthermore, 24 h-UCU-values depend on the dose of chelating agents. It has therefore been concluded that the role of 24 h-UCU is limited in therapy monitoring of WD (29).

Our experience is that patients may forget to collect the urine in time before a control visit, do not document the exact volume of the collected urine and do not bring the urine with them in specially prepared, suitable vessels (40). Our observations indicate and confirm the conclusion by Mohr and Weiss (2019) that the use of the 24 h-UCU-value for therapy monitoring is questionable (29). In our cohort of 108 WD-patients only nine patients could be detected with all their 24 h-UCU-values lower than 60 μg/d. But none of these nine patients had more than 7 measurements of 24 h-UCU. Thus, these patients had not been treated for a long time. Obviously, this criterion of compliance was too restrictive or the compliance of the WD-patients to collect the 24 h-urine was too low. On the other hand, in 29 of 108 patients all the 24 h-UCU-values were larger than 60 μg/d, but only 9 of these 29 patients had more than 7 measurements of 24 h-UCU-values. This criterion of non-compliance also appeared to be highly restrictive. In the majority of the patients (with less or more than eight measurements) 24 h-UCU-values larger and lower than 60 μg/d were found. Although we agree with Dziezyc et al. (12) that determination of 24 h-UCU after 48 h cessation of medication is helpful in therapy monitoring and reflects aspects of compliance, it seems to be difficult to develop criteria for compliance or non-compliance solely on the 24 h-UCU-values.

### 4.4 CHE-S as compliance assessment

The serum levels of butyrylcholinesterase (CHE-S) are low in untreated WD-patients and can be used to differentiate not only between WD-patients and normal subjects, but also between WD-patients and heterozygous gene carriers (28). Thus, CHE-S is a useful biomarker for untreated WD. Furthermore, it is a sensitive biomarker to measure liver recovery after onset of therapy in WD (11). Therefore, the hypothesis that CHE-S is lower in non-compliant than in compliant patients is difficult to test and to interpret during the first few years of WD-specific treatment. The value of CHE-S after a short time of therapy not only depends on compliance to therapy but also on the initial value of CHE-S at time of diagnosis.

Because of that reason we only compared compliant and non-compliant patients who had at least eight 24 h-UCU-values and had been treated for at least 2 years. As mentioned above separation of compliant and non-compliant patients solely on the basis of the 24 h-UCU-values was difficult. Nevertheless, significant lower CHE-S-values could be detected in thus-defined non-compliant compared to ermpliant patients. This was demonstrated for the last CHE-S-value determined at the day of recruitment as well as the mean CHE-value determined over all available measurements.

In contrast to CU-S, CER-S, and 24 h-UCU CHE-S was the only parameter with a mean value within the normal range. Thus, CHE-S varies over a large range during WD-specific therapy. It is easily available and cheap. In a subsequent study it will be demonstrated that in individual patients CHE-S may be even more appropriate to detect improvement and worsening of outcome in WD during the time course of therapy in WD than the gold-standard, the 24 h-urinary copper excretion.

## 5 Conclusions and clinical implications

CHE-S is modified over a wide range during specific long-term treatment in WD. It is highly significantly correlated with 24 h-UCU and is different in compliant and non-compliant patients. Because of its sensitivity to detect worsening and improvement of liver function during therapy we recommend that this easily available and cheap parameter is used in further studies and clinical practice to establish its value for therapy monitoring in WD.

## 6 Strengths and limitations of the study

The strength of the study is that the initial hypothesis could be confirmed, and that CHE-S turned out to be sensitive enough to discriminate between compliant and non-compliant patients in WD as had been demonstrated for the 24 h-urinary copper excretion previously (12). The use of 24 h-UCU-values for the definition of compliance in the present study, however, caused some difficulties which might not have occurred with structured interviews. Therefore, further multi-center studies are recommended to demonstrate the value of CHE-S as an assessment tool for compliance and patient management in WD.

A further limitation for the use of butyrylcholinesterase as compliance assessment in WD is its low specificity. The possibility exists that a genetically determined butyrylcholinesterase deficiency syndrome may also be present in a WD-patient (41) or another comorbidity causing liver dysfunction. Therefore low CHE-S-values have to be interpreted in concert with other laboratory findings.

## Data Availability

The raw data supporting the conclusions of this article will be made available by the authors, without undue reservation.

## Glossary

AGE: age at recruitment
AGO: age at diagnosis
ATP7B: P-adenotriphosphatprotease
AUC: area under the curve
CER: ceruloplasmin
CER-S: serum level of CER
CHE: butyrylcholinesterase
CHE-S: serum level of CHE
COM-NCC-group: group of compliant patients when classified according to NCC-S
COM8-group: group of compliant patients when classified according to 8 24 h-UCU values
CU: copper
CU-S: serum level of CU
DPA: D-penicillamine
DURT: duration of treatment
H-COM1: highly compliant patients when classified according to 1 24 h-UCU-value
IN-CHE-S: first CHE-S-value of a patient
LA-CHE-S: last CHE-S-value of a patient
L-COM1-group: less compliant patients when classified according to 1 24 h-UCU value
MV: mean value
NCC-S: calculated free non-ceruloplasmin bound serum copper
N-COM8-group: group of non-compliant patients when classified according to 8 24 h-UCU values
N-COM-NCC-group: group of non-compliant patients when classified according to NCC-S
N-COM1-group: group of non-compliant patients when classified according to 24 h-UCU
RCC: rang correlation coefficient
ROC-curve: receiver operating characteristics
SD: standard deviation
24 h-UCU: 24 h-urinary copper excretion
WD: Wilson’s disease
